# Malignant peripheral nerve sheath tumor of nasal cavity and paranasal sinus with 13 years of follow‐up—A case report and review of literature

**DOI:** 10.1002/ccr3.2465

**Published:** 2019-10-04

**Authors:** Toms Vengaloor Thomas, Anu Abraham, Eldrin Bhanat, Youssef Al Hmada, Ashley Albert, Srinivasan Vijayakumar, Scott P. Stinger, Satyaseelan Packianathan

**Affiliations:** ^1^ Department of Radiation Oncology University of Mississippi Medical Center Jackson Mississippi; ^2^ Department of Pathology University of Mississippi Medical Center Jackson Mississippi; ^3^ Department of Otolaryngology and Communicative Sciences University of Mississippi Medical Center Jackson Mississippi

**Keywords:** head and neck sarcoma, malignant peripheral nerve sheath tumors, Sarcoma of nasal cavity and para nasal sinus

## Abstract

Although extremely rare, sarcomas including malignant peripheral nerve sheath tumors should be considered in the differential diagnosis of sino‐nasal tract lesions. Long‐term cure is possible through definitive operative management followed by adjuvant therapy.

## Graphical abstract

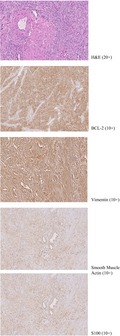

## 1 INTRODUCTION

Malignant peripheral nerve sheath tumors (MPNSTs) are extremely rare sarcomas of the nasal cavity and paranasal sinuses. We present a case report of a malignant peripheral nerve sheath tumor of the nasal cavity and paranasal sinus in an African American female with 13 years of follow‐up. The patient underwent resection of the tumor with operative pathology revealing an intermediate grade malignant peripheral nerve sheath tumor with close margins. She subsequently received adjuvant radiation to a total dose of 50.4 Gy using intensity modulation radiation therapy (IMRT). Although extremely rare, sarcomas including MPNSTs should be considered in the differential diagnosis of sino‐nasal tract lesions. Long‐term cure is possible through definitive operative management followed by adjuvant therapy.

Sarcomas of the head and neck region are relatively rare entities, accounting for only 1% of all malignancies in that region.^1^ Five percent of head and neck sarcomas present in the nasal cavity or paranasal sinuses.^1^ Location in the nasal cavity portends a worse prognosis.^2^ MPNSTs are particularly rare. Correspondingly, a MPNST of the head and neck region is very rare^3^ and even rarer when located in the nasal cavity or paranasal sinuses.^4^ Here, we report on a case of a MPNST of the nasal cavity in a patient who has no evidence of disease after treatment and 13 years of follow‐up. We review the literature and the similar cases that have been reported.

## 2 CASE

### 2.1 Presentation

A 47‐year‐old African American woman presented to the clinic on July 2006 with a 1‐year history of left nasal obstruction and intermittent epistaxis. Her past medical history was significant for hypertension, hyperlipidemia, and coronary artery disease all of which were appropriately managed. She had no history of prior malignancy or immunosuppression or neurofibromatosis.

### 2.2 Workup

**Figure 1:**
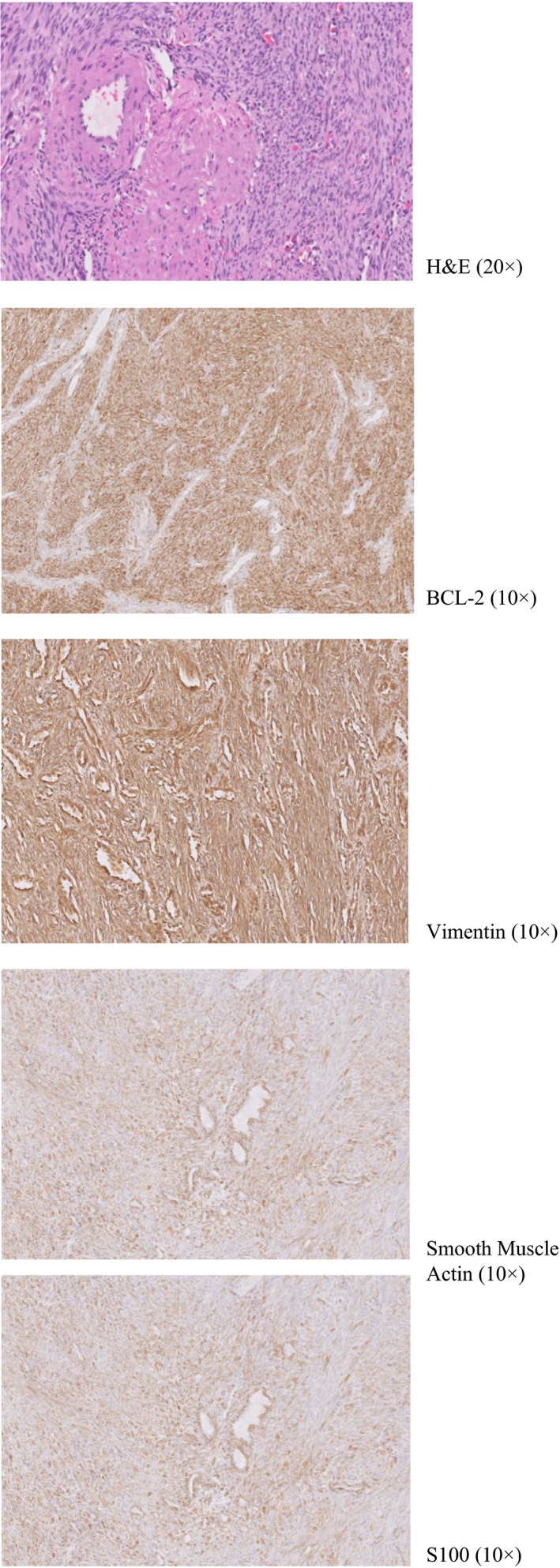
A‐E, Pathology: Spindle cell proliferation arranged in short fascicles with some hyalinized whorls. The lesion is very cellular with few mitoses and warrants an intermediate grade. No necrosis or significant pleomorphism is seen. The neoplastic cells are strongly positive for smooth muscle actin, S‐100, BCL‐2, and vimentin

**Figure 2:**
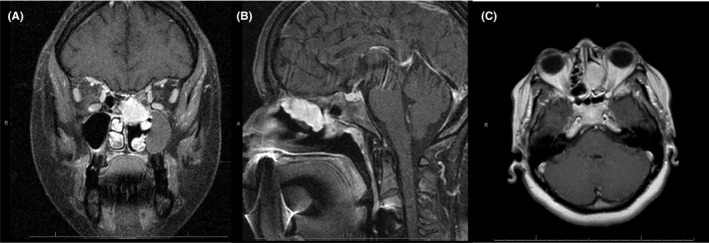
A‐C, MRI at diagnosis. The 2.1 × 1.7 × 3.5 cm mass in the superior left nasal cavity demonstrates avid enhancement. There is no MRI evidence of perineural tumor extension, intracranial extension into the cribriform plate, or cavernous sinus involvement

She underwent computed tomography (CT) scan of the sinuses which revealed a left nasal mass measuring 3.4 × 3.1 × 2.4 cm with associated thinning of the medial orbital wall, nasal septum, and medial wall of the maxillary sinus. A biopsy of the mass was obtained and reported as a MPNST, intermediate grade. Tissue staining was positive for S100 but negative for EMA, AE1/AE3, and Desmin (Figure 1A‐E). A subsequent magnetic resonance imaging (MRI) scan of the brain was obtained for further evaluation and surgical planning (Figure 2A‐C). A CT scan of the chest did not reveal any distant metastatic disease.

### 2.3 Treatment

Neurosurgery and otolaryngology performed an anterior craniofacial extradural skull base resection, bilateral endoscopic ethmoidectomies, right sphenoidectomy, right maxillary antrostomy, and left maxillary sinus antrostomy with removal of contents. The postoperative period was uneventful, and patient recovered well. Final pathologic assessment confirmed an intermediate grade MPNST and negative surgical margins (R0 resection).

After review at multidisciplinary head and neck tumor board, she underwent adjuvant radiation therapy to the postoperative bed to a total dose of 5040 cGy in 28 fractions. A 7‐field IMRT treatment plan with daily image guidance was utilized in her treatment.

Six months following the radiation therapy, she underwent lysis of synechiae that was causing obstruction of the left nasal cavity as well as submucosal resection of the bilateral inferior turbinate—pathology was benign. She also underwent debridement of her craniofacial plate and drainage of an epidural abscess a few months later—again, the pathology from this procedure did not show any evidence of disease persistence or recurrence. She has since done well and continues on yearly surveillance with flexible nasopharyngolaryngoscopy and cross‐sectional imaging using MRI scan. Thirteen years after completion of her treatment, she demonstrates no clinical evidence of disease.

## 3 REVIEW OF CASES IN THE LITERATURE

We conducted a literature review using the following key words: nasal sarcoma, sarcoma of the nasal cavity and paranasal sinus, head and neck sarcoma, malignant peripheral nerve sheath tumor, and MPNST of nasal cavity. MPNST was previously known as neurofibrosarcoma, neurosarcoma, or malignant schwannoma; so, we added these terms to the literature search, even though these terms are considered obsolete.^5^ Malignant triton tumors are MNPST with a component of rhabdomyoblastoma.^5^, ^6^ There are only a small number of these cases reported in the literature, and they are documented in Table 1.^7^, ^8^, ^9^, ^10^, ^11^, ^12^, ^13^, ^14^, ^15^, ^16^, ^17^, ^18^, ^19^, ^20^, ^21^, ^22^, ^23^ All these are single case reports, except a case series reported by Hellquist et al,^19^ which contained five cases. Most of these, however, have only short duration follow‐up.

**Table 1:** Nasal cavity/para nasal cavity Malignant peripheral nerve sheath tumor (MPNST) cases reported in the literature

Author	No	Subsite	NF	Pathology	Grade	Treatment	Adjuvant RT	Adjuvant chemo	Follow‐up	Local control	Survival
Wang et al^10^	1	Maxilla	No	MPNST	N/A	Total maxillectomy	Yes	No	1 yr	Yes	Yes
Nagayama et al^11^	1	Maxilla	No	Malignant schwannoma	G3	Total maxillectomy, orbital, and ethmoid exenteration	No	No	3 yrs	Yes	Yes
Robitaille et al^13^	1	Maxilla	Yes	Malignant schwannoma	N/A	Maxillectomy	RT at recurrence	No	7 ms	No	No
Purohit et al^8^	1	Nasal cavity	No	Malignant schwannoma	N/A	Lateral rhinotomy with removal of tumor	No	No	3 yrs	Yes	Yes
Lee et al^9^	1	Frontal, ethmoidal, and orbit	Yes	MPNST	N/A	Wide resection	No	No	6 ms	Yes	Yes
Padua et al^12^	1	Nasal cavity, maxillary, ethmoid, and sphenoid	No	Malignant schwannoma	N/A	Wide resection by lateral rhinotomy	Yes	No	2 yrs	Yes	Yes
Das et al^18^	1	Nasal cavity, maxillary sinus, and orbit	No	MPNST	N/A	Medial maxillectomy and resection of the orbital extension of tumor	No	Yes	2 yrs	Yes	Yes
Hellquist et al^19^	5	1. Nasal cavity/ ethmoids	No	Neurogenic sarcoma	N/A	1. Radical surgery using Denker approach	No	No	8 m	Yes	Yes
		2. Nasal cavity, ethmoids, sphenoid, and anterior cranial fossa	No	Neurogenic sarcoma	N/a	2. Combined intra and extracranial surgery	No	No	7 m	Yes	Yes
		3. Maxilla/hard palate	No	Neurogenic sarcoma		3. Maxillectomy; Recurrence was resected 1 y later	No	No	17 yrs	Yes	Yes
		4. Maxilla	No	Neurogenic sarcoma	N/a	4. Maxillectomy and ethmoidectomy	No	No	3 yrs	No	No
		5. Maxilla/Hard palate	No	Neurogenic sarcoma		5. Maxillectomy	No	No	6 yrs	Yes	Yes
Ogunleye et al^7^	1	Nasal cavity, frontal, ethmoidal sinus, orbit, and anterior cranial fossa	No	Malignant schwannoma		Wide resection	Yes	No	N/a	Yes	N/a
Goswami et al^15^	1	Nasal cavity and ethmoid sinus	No	Neurofibrosarcoma	G1	Lateral rhinotomy and wide resection of tumor	No	No	6 mo	Yes	Yes
Osuch‐Wojcikiewicz et al^15^	1	Nasal cavity and maxillary sinus	No	Neurosarcoma	N/A	Lateral rhinotomy and wide local resection	RT at recurrence	No	3.5 y	No	No
Lee et al^17^	1	Nasal cavity and septum	No	Malignant schwannoma	N/A	Endoscopic excision	Yes	No	1 y	Yes	Yes
Ahsan et al^20^	1	Nasal cavity and PNS	No	MPNST	N/A	Wide resection	Yes	No	No	Yes	No
Pan et al^21^	1	Nasal cavity and nasopharynx	No	MPNST	N/A	Endoscopic excision of the mass	No	Yes	9 mo	Yes	Yes
Schick et al^22^	1	Frontal sinus	No	MPNST	N/A	Endonasal resection					
Pfeiffer et al^23^	1	Nasal cavity, ethmoid, and sphenoid	No	MPNST	N/A	Radical resection with lateral rhinotomy Trans frontal resection of the skull bas tumor at recurrence	No	No	45 m	Yes	Yes

The majority of the MPNSTs of the nasal cavity and paranasal sinuses reported thus far in the literature were not associated with neurofibromatosis. Most of the patients were managed with definitive surgery using a lateral rhinotomy or endoscopic approach depending upon the extent of their disease. Many of the patients received adjuvant radiation treatment but none received neoadjuvant radiation therapy. Adjuvant chemotherapy use was reported in two patients.

## 4 DISCUSSION

### 4.1 Epidemiology

Head and neck cancer is common and is the sixth most common cancer globally. Among the different histologies of head and neck cancers, sarcomas are extremely rare, accounting for only ~1% of all the malignancies in the region^1^; moreover, they represent only about 5%‐10% of the malignancies of the entire body.^24^ The majority (up to 70%) of the sarcomas involve skin and soft tissue; the nasal cavity and paranasal sinus location represents only about 5% of all head and neck sarcomas.^1^, ^2^, ^4^

Sarcomas are cancers arising from cells of mesenchymal origin. Sarcomas account for ~1% of adult malignancies with almost 13 000 new cases expected and 5000 deaths anticipated in 2019.^25^, ^26^ Almost 50 different histologic types of sarcoma have been reported so far.^5^ The MPNST is a relatively rare entity and accounts for only 5%‐10% of sarcomas^27^, ^28^ with an incidence of 0.001% in the general population. This disease is more common in patients with neurofibromatosis 1 (NF1), and the lifetime risk of developing MPNST in such patients is up 10%.^27^, ^28^ There have only been a few case reports of MPNST arising from nasal cavity and paranasal sinuses in the literature.

### 4.2 Pathology

Malignant peripheral nerve sheath tumors are usually large lesions that may sometimes cause a fusiform expansion of the nerve from which they arise.^5^, ^29^ Depending upon the stroma and cellularity, the tumors can be fibrous, gelatinous, or fleshy in consistency. Grossly, MPNSTs appear as large masses, producing fusiform enlargement of major nerves. Histologically, most MPNSTs are composed of highly cellular fascicles of spindle cells, sometimes with a vaguely whorled growth pattern. The spindle cells in MPNST are typically uniform, with palely eosinophilic cytoplasm and indistinct cell borders, and hyperchromatic thin nuclei, with wavy or focally buckled shapes. There is often some degree of nuclear pleomorphism. In the more frequent intermediate‐ and high‐grade tumors, mitotic figures are often readily identified. However, low‐grade MPNSTs may show very scarce mitotic activity. Some specimens show uniformly high cellularity throughout the tumor, with a fibrosarcoma‐like fascicular growth pattern similar to monophasic synovial sarcoma. More often though, tumors are composed of relatively hypocellular areas alternating with hypercellular areas showing perivascular accentuation, resulting in a marbled appearance at low magnification. The extracellular matrix in less cellular areas is usually myxoid, which may be abundant in up to 10% cases. Clusters of small, rounded blood vessels are commonly seen in high‐grade tumors.

Loss of H3K27me3 staining is observed in 50% of MPNSTs overall, including 30% of the low‐grade tumors and up to 90% of the high‐grade MPNSTs. About half of the tumors express S‐100 protein, typically in only a focal or patchy distribution, although the low‐grade MPNSTs arising in a neurofibroma may show more consistent staining. GFAP and SOX10 are positive in 30%‐40% of cases. CD34 is often positive, sometimes extensively. EMA may show focal staining, and focal desmin expression is not uncommon.

### 4.3 Clinical evaluation

Early diagnosis is the key to successful treatment, leading to longer survival. There are no screening guidelines available for nasal cavity mass lesions. A clinical diagnosis of mass lesion in nasal cavity should be confirmed by biopsy. MRI with and without contrast of the head and neck is recommended as it more accurately determines the relationship of the tumor with the surrounding neurovascular bundles and muscles, and thus will assist in surgical and radiation treatment planning.^25^, ^30^ A CT scan of the chest with and without contrast or a PET‐CT scan is recommended for the metastatic evaluation.^31^

### 4.4 Differential diagnosis

The differential diagnosis for nasal cavity masses includes nasal polyps, mucoceles, Schneiderian papillomas, and malignancies.^32^ Malignancies of the nasal cavity can include squamous cell carcinoma, adenocarcinoma and its variants, neuroendocrine carcinoma (also known as olfactory neuroblastoma or esthesioneuroblastoma), and melanoma.

### 4.5 Staging

In the new American Joint Committee on Cancer (AJCC), eighth edition, the staging for head and neck sarcomas has been changed.^33^

### 4.6 Treatments

#### 4.6.1 Surgical management

The definitive management of sarcomas of the head and neck is surgical resection with appropriate margins.^25^ In head and neck region, wide margins may not always be possible due to the proximity of critical structures, neurovascular bundles, or for cosmetic reasons. Resections can result in close or even positive margins. If margins are grossly positive, re‐resection should be considered. If re‐resection is not feasible, adjuvant radiation therapy should be administered, with cone‐down boost dose to the areas of grossly positive margins.

#### 4.6.2 Radiation treatments

Radiation treatment can be considered pre‐ or postoperatively for sarcoma treatment—each approach with its own pros and cons. The potential benefits of pre‐operative radiation include lower radiation dose, shorter treatment course, smaller treatment fields, and potential downstaging of the tumor.^25^ The advantage of a postoperative radiation paradigm includes a lower rate of postoperative complications and the possibility that radiation can be avoided if the final margins are widely negative. Radiation treatment confers an improvement in local control, but not overall survival.^34^, ^35^, ^36^ However, excellent survival rates have been reported after definitive or postoperative radiation treatment for sarcoma of the head and neck region.^36^, ^37^

The role of pre‐operative radiation treatment in head and neck sarcoma is not well studied. In a report from Princess Margaret Hospital, O’ Sullivan et al reported that pre‐operative radiation treatment to 50 Gy was well tolerated and provided a high rate of local control. They also reported lower rates of major wound complications as compared with extremity sarcomas (20% vs 35%). There are few case reports on pre‐operative radiation for HN sarcomas^38^ as well, but radiation treatment is typically delivered after definitive operative management in the head and neck region as its use depends upon the surgical margins and pathologic diagnosis.

External beam radiation treatment (EBRT) is most commonly used in the treatment of sarcoma. The accepted pre‐operative dose is 50 Gy, and a postoperative boost may be considered if the margins are positive. The boost treatment can be delivered using EBRT, brachytherapy, or intra operative radiation therapy (IORT).^25^ In the adjuvant setting, the dose will typically be 50 Gy followed by a boost of at least 10 Gy, which can vary depending upon the margin status. IMRT provides better target coverage and can improve several of the side effects of treatment.

#### 4.6.3 Chemotherapy

The role of concurrent chemotherapy with pre‐operative or postoperative radiation treatment is not well established in patients with head and neck sarcomas. Pre‐operative chemoradiation has been used for high‐grade, high‐risk extremity sarcomas and appears to improve local control and overall survival, but has significant treatment‐related toxicity as well.^39^, ^40^ For the high‐grade sarcomas, there may be a benefit from the delivery of adjuvant chemotherapy.^41^ In the metastatic setting, palliative chemotherapy is the treatment of choice, but local therapy may be considered for palliation, especially in the head and neck region.

### 4.7 Surveillance

Patients with neurofibromatosis 1 (NF1) are at higher risk of developing MPNSTs in other areas as well as gastrointestinal stromal tumors (GIST). Hence, they need closer monitoring so that any malignancy can be identified early; whole‐body MRI scans can be considered.^25^

## 5 CONCLUSION

Malignant peripheral nerve sheath tumors (MPNSTs) of the nasal cavity and paranasal sinuses are extremely rare entities due to a combination of rarity of the type of the cancer and its rarity at the location cited. Even though the incidence is extremely rare, sarcomas should be considered in the differential diagnosis for sino‐nasal tract lesions. Long‐term cures are possible through definitive operative management followed by adjuvant treatment(s) depending on tumor grade and margin status.

## CONFLICT OF INTEREST

None declared.

## AUTHORS CONTRIBUTIONS

All authors contributed to implementation, monitoring, and research on described methodology.

## ETHICAL APPROVAL

Institutional review board, University of Mississippi Medical Center.

## CONSENT FOR PUBLICATION

Not applicable.

## Data Availability

Please contact author for data requests.
